# Differential depression of neuronal network activity by midazolam and its main metabolite 1-hydroxymidazolam in cultured neocortical slices

**DOI:** 10.1038/s41598-017-03154-5

**Published:** 2017-06-14

**Authors:** Monika Balk, Harald Hentschke, Uwe Rudolph, Bernd Antkowiak, Berthold Drexler

**Affiliations:** 1 Department of Anaesthesiology, Experimental Anaesthesiology Section, Eberhard-Karls-University, Waldhörnlestrasse 22, 72072 Tübingen, Germany; 2 Laboratory of Genetic Neuropharmacology, McLean Hospital and Department of Psychiatry, Harvard Medical School, 115 Mill Street, Belmont, MA 02478 USA; 3 Department of Psychiatry, Harvard Medical School, 401 Park Drive, Boston, MA 02215 USA; 4 Werner Reichardt Centre for Integrative Neuroscience, Eberhard-Karls-University, Otfried-Müller-Str. 25, 72076 Tübingen, Germany; 5 Department of Anaesthesiology and Intensive Care Medicine, Esslingen Hospital, Hirschlandstr. 97, 73730 Esslingen, Germany

## Abstract

The benzodiazepine midazolam is widely used in critical care medicine. Midazolam has a clinically active metabolite, 1-hydroxymidazolam. The contribution of 1-hydroxymidazolam to the effects of midazolam is controversial. The aim of the current study was to compare the actions of midazolam and 1-hydroxymidazolam on network activity of cortical neurons. Midazolam depressed neuronal activity at a low concentration of 5 nM. When midazolam concentration was increased, it depressed neuronal discharge rates in a biphasic manner. In comparison, 1-hydroxymidazolam did not depress the cortical network activity at low nanomolar concentrations. Higher concentrations of 1-hydroxymidazolam consistently inhibited neuronal activity. Moreover, midazolam shortened cortical up states at low, but not at high concentrations, while the opposite effect was observed with 1-hydroxymidazolam. The network depressant action of midazolam at low concentrations was absent in slices from GABA_A_ receptor α_1_(H101R)mutant mice. The α_1_(H101R)mutation renders α_1_-subunit containing GABA_A_ receptors insensitive towards benzodiazepines. This GABA_A_ receptor subtype is thought to mediate sedation. As midazolam is more potent than its metabolite 1-hydroxymidazolam, the major clinical effects are thus likely caused by midazolam itself. However, 1-hydroxymidazolam could add to the effects of midazolam, especially after the application of high doses of midazolam, and in case of impaired drug metabolism.

## Introduction

Midazolam is a commonly used benzodiazepine in anaesthesia and intensive care medicine. Midazolam has a clinical active metabolite: 1-hydroxymidazolam, which is, like the parent drug midazolam a neuronal depressant drug. However, 1-hydroxymidazolam’s contribution to the depression of neuronal activity and thus its clinical relevance still remains a matter of debate. There are numerous studies in which the statements concerning the contribution of 1-hydroxymidazolam to the clinical actions of midazolam range from “almost equipotent” to “no major contributing factor”^1–5^. In addition, Crevoisier *et al*. have demonstrated that cognitive and motor impairments induced by midazolam are different after oral or intravenous application, an observation that was attributed to the relatively higher amount of 1-hydroxymidazolam after oral administration^6^.

The current study was therefore designed to directly compare the effects of midazolam and 1-hydroxymidazolam on the activity of neocortical neurons in organotypic slice cultures, a model system in which, to the best of our knowledge, there is no relevant metabolism from midazolam to 1-hydroxymidazolam.

Basically, benzodiazepines like midazolam predominantly act via GABA_A_ receptors harbouring either an α_1_, α_2_, α_3_ or α_5_-subunit. About 40% of all GABA_A_ receptors in the brain contain α_1_-subunits, making them the major GABA_A_ receptor subtype in the brain^7^. Mice carrying a point mutation in the α_1_ subunit of the GABA_A_ receptor at position 101 are resistant to the sedative action of diazepam, indicating that benzodiazepine-induced sedation is predominantly mediated via GABA_A_ receptors containing the α_1_-subunit^8, 9^. Moreover, it was suggested that sedation with diazepam depends on GABA_A_ receptors located on glutamatergic neurons in the cerebral cortex^10^. On the cellular level, this molecular action of diazepam translates into a significant depression of cortical network action potential firing^11^. Therefore, in order to examine the contribution of α1-containing GABA_A_ receptors to this phenomenon, in the current study clinically relevant concentrations of midazolam were also tested in neocortical slice cultures from GABA_A_ receptor α_1_(H101R) knock-in mice.

## Results

### Neuronal activity of organotypic neocortical slice cultures under control conditions

**Figure 1:**
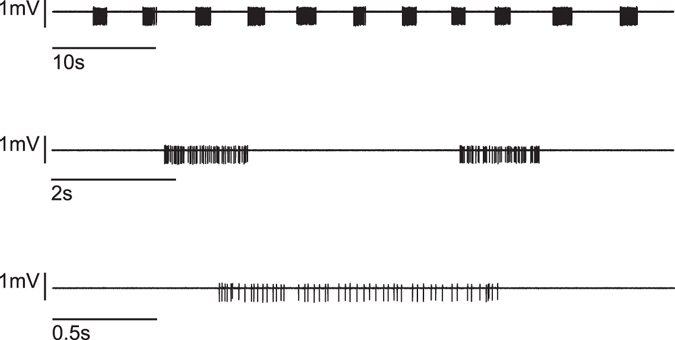
Original recording from a neocortical slice culture. An extracellular recording from an organotypic murine slice culture is displayed at three different time scales. The recorded signal was band-pass filtered (200–2000 Hz) to separate action potentials from local field potentials. Upper row: One minute recording of neuronal activity: eleven up states (phases of high frequency action potential firing) are separated by neuronal silence (down states). Middle row: Ten seconds of the trace given above at higher temporal resolution. Two up states can be identified, halted by neuronal silence. Lower trace: a single up state at high temporal resolution, single action potentials can be identified.

Spontaneous firing patterns from neocortical organotypic slice cultures were characterized by phases of high neuronal activity (up states) separated by periods of neuronal silence. A typical extracellular recording is displayed in Fig. 1. To analyse changes of discharge rates in the presence of drugs we used the following parameters: i) the action potential firing rate and ii) the length of up states. Up states of neocortical slice cultures are characterized by high frequency action potential firing at the beginning of the up state, which is then gradually decreasing over time^12^. The median of the spontaneous action potential firing rate (in C57/BL6J mice) was at 10.12 Hz; interquartile range (iqr): 12.92; (number of recordings n = 481).

### Effects of midazolam in organotypic slice cultures (C57BL/6J)

**Figure 2:**
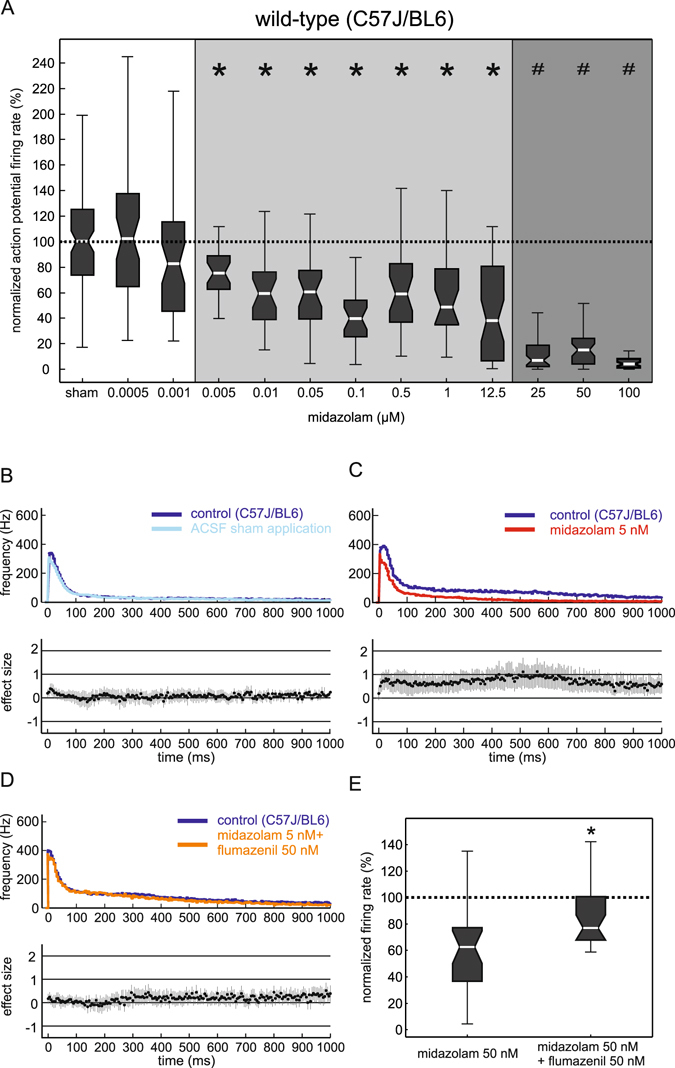
The effects of midazolam on global neuronal activity. (**A**) Midazolam inhibited the action potential firing rate of neocortical cultures in a biphasic manner. Over a wide concentration range starting at 5 nM up to 12.5 μM the residual activity roughly averaged 60% (*p < 0.001 compared with ACSF sham application, light grey). When increasing the concentration to 25 μM a further depression was observed, statistically different compared with the former concentration range (^#^p < 0.001, dark grey) (n/concentration: 27–50). All data are normalized to the median of the ACSF sham application. The dotted line marks 100%, i.e. no difference compared to ACSF sham application (statistical analysis: Kruskal-Wallis test with post-hoc analysis (Bonferroni) and Mann-Whitney U test for testing between two different groups). (**B**–**D**) Effects of midazolam on neuronal up states displayed as peri-event time histogram (PETH). To illustrate the differences, the effect size (Hedges’ g including the 95% confidence interval) is given below the PETH. Effects can be regarded as significantly different, if the 95% confidence interval does not cross the zero line. (**B**) Time-dependant changes of neuronal activity under control condition (dark blue) and ACSF sham application (light blue). Besides a negligible reduction of neuronal activity at around 15 ms after the beginning of the up state, the action potential firing did not change over time during ACSF sham application. (**C**) Actions of midazolam at a concentration of 5 nM (red, n = 28) versus control condition (dark blue). The effect size diagram illustrates that 5 nM midazolam continuously induced a significant inhibition during the first 1000 ms of the up state. (**D**) Effect of midazolam at a concentration of 5 nM in the additional presence of flumazenil (50 nM, orange, n = 28) versus control condition (dark blue). The effect size diagram displays that the significant inhibition during the first 1000 ms of the up state is absent in the presence of flumazenil. (**E**) Left box plot shows the normalized action potential firing in the presence of 50 nM midazolam alone. Right box plot shows the normalized firing rate after the combined application of 50 nM midazolam and 50 nM flumazenil (*p < 0.001).

Midazolam in a concentration range from 5 nM to 100 µM inhibited the neuronal firing rate in a biphasic manner. From 5 nM to 1 µM, midazolam significantly depressed action potential firing. However, the inhibitory action of midazolam did not rise continuously with increasing concentrations. Actually, 500 nM and 1 µM midazolam depressed the neuronal activity only slightly less than the administration of 100 nM midazolam. Though, raising the concentration of midazolam to 25 µM and beyond led to a stronger depression, which was significantly different from the concentration range of 5 nM to 1 µM (Fig. 2A, Kruskal-Wallis test and post-hoc Bonferroni analysis).

Even a low concentration of 5 nM midazolam induced a significant change in neuronal activity (median 76%, iqr 26%; p < 0.001; n = 28; Mann-Whitney U test).

To further elucidate the actions of midazolam at 5 nM we calculated a peri-event time histogram (PETH) for the first 1000 ms of the average up state. The sham application of ACSF only led to negligible changes at the beginning of the up states (Fig. 2B). By comparison, 5 nM midazolam consistently inhibited action potential firing significantly over the entire first 1000 ms of the average up state (Fig. 2C).

To test whether this action of midazolam is mediated via the classical benzodiazepine binding site of the GABA_A_ receptor, we performed experiments with flumazenil, a selective antagonist at the benzodiazepine binding site of the GABA_A_ receptor^13, 14^. As shown in Fig. 2D,E the network depressing actions of midazolam (5 nM and 50 nM) indeed proved to be almost completely absent when administering 50 nM flumazenil.

### Effects of 1-hydroxmidazolam in organotypic cultures and comparison to those of midazolam

To compare the actions of midazolam with those of 1-hydroxymidazolam we tested a concentration range of 1-hydroxymidazolam from 5 nM to 1 µM, because the *in vivo* concentration range of 1-hydroxymidazolam is lower than that of midazolam itself ^3^.

**Figure 3:**
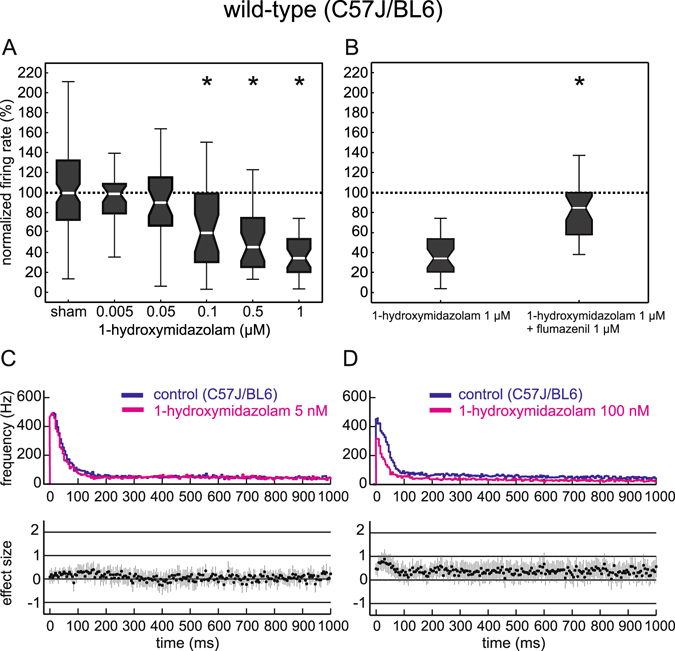
Effects of 1-hydroxymidazolam in neocortical slice cultures from wild-type mice. (**A**) Effects of 1-hydroxymidazolam on cortical network activity. 1-hydroxymidazolam did not induce significant changes in the neuronal action potential firing rate at low concentrations (5 nM and 50 nM). Only at concentrations ranging from 100 nM to 1 μM a significant inhibition of the action potential firing rate could be observed. Note that the depressing effect steadily rose by increasing the concentration of 1-hydroxymidazolam (*p < 0.001; Kruskal-Wallis test, post-hoc analysis (Bonferroni) followed by Mann-Whitney U test). (**B**) Actions of 1-hydroxymidazolam in the presence of the benzodiazepine antagonist flumazenil. Left box plot shows the normalized firing rate of 1 μM 1-hydroxymidazolam, right box plot shows the normalized firing rate after the combined application of 1 μM 1-hydroxymidazolam and 1 μM flumazenil (*p < 0.001). (**C**) PETH showing the effects of 5 nM 1-hydroxymidazolam (magenta, n = 34) versus control condition (dark blue). Unlike midazolam, 1-hydroxymidazolam did not depress neuronal activity during up states at low concentrations. For the effect of midazolam at the corresponding concentration please see Fig. 1C. (**D**) Actions of 100 nM 1-hydroxymidazolam (magenta, n = 34), versus control condition (dark blue) given as PETH.

The concentration response relationship for 1-hydroxymidazolam is displayed in Fig. 3A. In contrast to midazolam, we could not find an inhibitory effect for 1-hydroxymidazolam at 5 nM and 50 nM. Only at concentrations of 100 nM or higher 1-hydroxymidazolam did induce a significant inhibition (median 60%, iqr 69; p < 0.001). Again, unlike midazolam, 1-hydroxymidazolam caused a steady increase in the depression of network activity with increasing concentrations of up to 1 µM (median 34%; iqr 33; p < 0.001).

To test whether the effect of 1-hydroxymidazolam is also mediated by the classical benzodiazepine binding site, we measured the network activity after administering a combination of 1 µM 1-hydroxymidazolam with 1 µM flumazenil. The depression of neocortical activity triggered by 1-hydroxymidazolam was almost completely antagonized by flumazenil (Fig. 3B).

**Figure 4:**
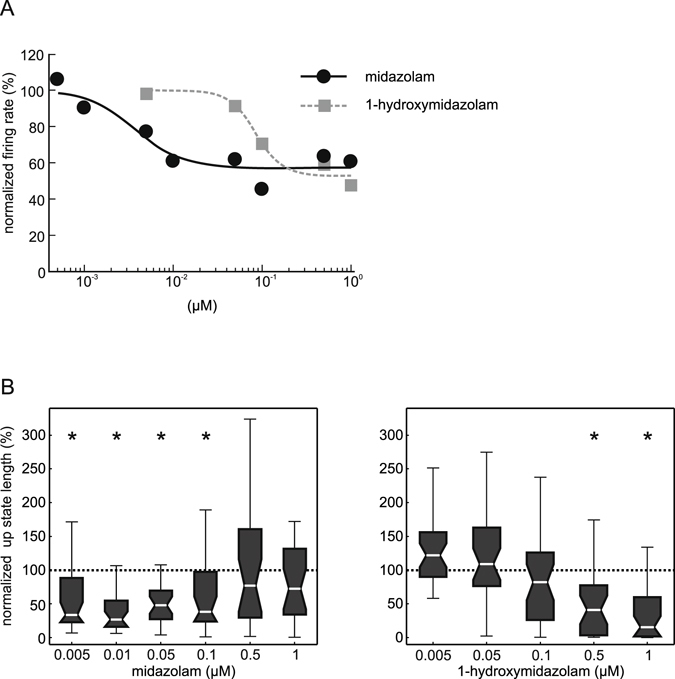
Comparison of the effects of midazolam and 1-hydroxymidazolam in neocortical slice cultures from wild-type mice. (**A**) Combined presentation of the effect of midazolam (black, taken from Fig. 1A) and 1-hydroxymidazolam (grey, taken from Fig. 2A) on the normalized action potential firing rate for comparison of actions. Data were fit as described in the methods section. Measures of statistical dispersion have been omitted for clarity reasons. Note that the two curves are shifted by roughly one order of magnitude. (**B**) Actions of midazolam and 1-hydroxymidazolam on the duration of phases of high neuronal activity (up state length). Data were also normalized to the median of the ASCF sham application (given by the dashed line). Midazolam significantly reduced the up state length between 5 nM and 100 nM. However, this effect was absent at 500 nM and 1 μM midazolam (*p < 0.001; Kruskal-Wallis test, post-hoc analysis (Bonferroni)). In comparison, 1-hydroxymidazolam significantly shortened the up state length starting at 500 nM (Kruskal-Wallis test, post-hoc analysis (Bonferroni)).

To further explain the quantitative differences of action between the two compounds in detail, we calculated PETHs for 1-hydroxymidazolam. As can be seen in Fig. 3C, 1-hydroxymidazolam did not show any inhibitory effect at a concentration of 5 nM. At a higher concentration (100 nM), 1-hydroxymidazolam was able to depress cortical activity, but its effect was only significant during the first 100 ms of the up states (Fig. 3D). A comparison of the actions of the parent drug midazolam and its metabolite 1-hydroxymidazolam is displayed in Fig. 4. Due to the biphasic nature of midazolam’s concentration response curve, we compared only concentrations up to the border of the wide ‘plateau’ region of the concentration response curves (1 µM of both midazolam and 1-hydroxymidazolam). As can be seen in the plot (Fig. 4A), IC_50_ values of both concentration response curves differed by over an order of magnitude (IC_50_ in nM with 95% CI: midazolam, 3.6 [0.9 6.4]; 1-hydroxymidazolam: 84.2 [48.7 119.8]; extra sums-of-squares test (see Methods for details): F(2,242) = 11.98, p = 8.6 * 10^−6^).

To further investigate the differences in action of midazolam and 1-hydroxymidazolam we analysed the length of up states. Under control conditions, the median length of up states was 664 ms (iqr: 2400). When midazolam was administered at low concentrations, it shortened the length of up states, while at 500 nM and 1 µM this effect was completely absent. On the other hand, 1-hydroxymidazolam only reduced the length of up states significantly at 500 nM and 1 µM (Fig. 4B). It is obvious that midazolam and 1-hydroxymidazolam affected up state length in quite different ways: while midazolam shortened up state length particularly at concentrations below 0.5 µM, 1-hydroxymidazolam did so only at concentrations above 0.1 µM (Fig. 4B). Statistical analysis (see Methods) confirmed this impression (two-way ANOVA of Box-Cox transformed data, interaction effect: F(4,289) = 14.2, p = 1.4 * 10^−10^, eta squared with 95% CI = 0.15 [0.09 0.25]).

**Figure 5:**
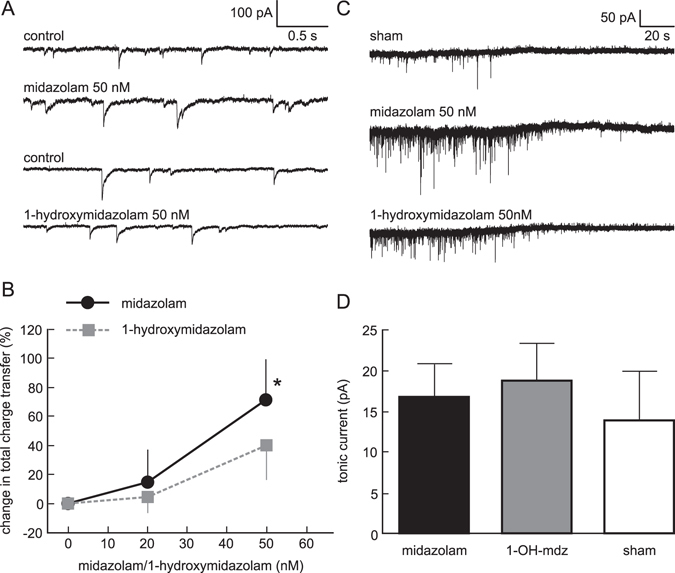
Effects of midazolam and 1-hydroxymidazolam on phasic and tonic currents of cultured cortical neurons. (**A**) Raw traces from two example whole cell recordings in neocortical neurons are displayed. Upper two traces: Control condition and presence of 50 nM midazolam. Midazolam induced a slight broadening, a slight increase in amplitude and a slight increase in frequency of IPSCs. Lower two traces: Control condition and presence of 50 nM 1-hydroxymidazolam. The effect of 1-hydroxymidazolam resembles the combined effect of midazolam, but overall was less pronounced. (**B**) Summary of the actions of midazolam and 1-hydroxymidazolam on IPSCs. The total charge transfer was calculated as area under the curve of the averaged IPSC multiplied by the frequency of IPSCs, normalized to control condition. At 50 nM the effect of midazolam on the total charge transfer is significantly different from sham application (n = 11, p = 0.046), while the effect of 1-hydroxymidazolam is not (n = 15, p = 0.022). (**C**) Raw traces from whole cell recordings illustrating the actions of midazolam and 1-hydroxymidazolam on tonic currents. Each recording shows 180 s starting at the application of 20 µM bicuculline. Top trace: Sham (ACSF) application plus bicuculline. Middle trace: Application of 50 nM midazolam plus bicuculline. Lower trace: Application of 50 nM 1-hydroxymidazolam plus bicuculline. Note that in each case a small tonic current is visible in the presence of bicuculline. (**D**) Summary of the actions of midazolam and 1-hydroxymidazolam on tonic inhibition. Both, midazolam and 1-hydroxymidazolam at a concentration of 50 nM induced a tonic current in cultured cortical neurons in the presence of 20 µM bicuculline. However, tonic currents by midazolam (n = 9) and 1-hydroxymidazolam (n = 12) were not significantly different from sham application (n = 7).

To further elucidate the actions of midazolam and 1-hydroxymidazolam at the synaptic level, we performed patch-clamp recordings in neocortical slice cultures from wild-type C57BL/6J mice. In a first set of experiments the effects on phasic inhibition were tested by recording miniature IPSCs from pyramidal neurons. To quantify drug actions on synaptical inhibition we used the total charge transfer, calculated as the area under the averaged IPSC multiplied by the frequency of IPSCs. Midazolam at a concentration of 50 nM increased total charge transfer by 71.6 ± 27.7% (n = 11, p = 0.045, t-test compared to sham application). At the same concentration, 1-hydroxymidazolam appeared to increase total charge transfer to a lesser extent, but this effect was not significant (40.4 ± 24.3%, n = 15, p = 0.22, t-test compared to sham application, see Fig. 5A,B). In a second set of experiments the actions of midazolam and 1-hydroxymidazolam on tonic inhibition were studied. For this purpose, neurons were additionally perfused with 20 µM bicuculline. Midazolam (50 nM) induced a tonic current of 16.87 ± 4.04 pA (n = 9, Fig. 5C,D), whereas 50 nM of 1-hydroxymidazolam induced a tonic current of 18.81 ± 4.57 pA (n = 12). However, both values were not significantly different from the basic tonic current induced in the presence of bicuculline and sham application (14.04 ± 5.87 pA, n = 7, one-way ANOVA, Fig. 5C,D).

### The role of α_1_-subunit containing GABA_A_ receptors for the actions of midazolam

Previous studies showed that cortical GABA_A_ receptors containing α_1_-subunits are an important target in mediating benzodiazepine-induced sedation^9, 10, 15^.

**Figure 6:**
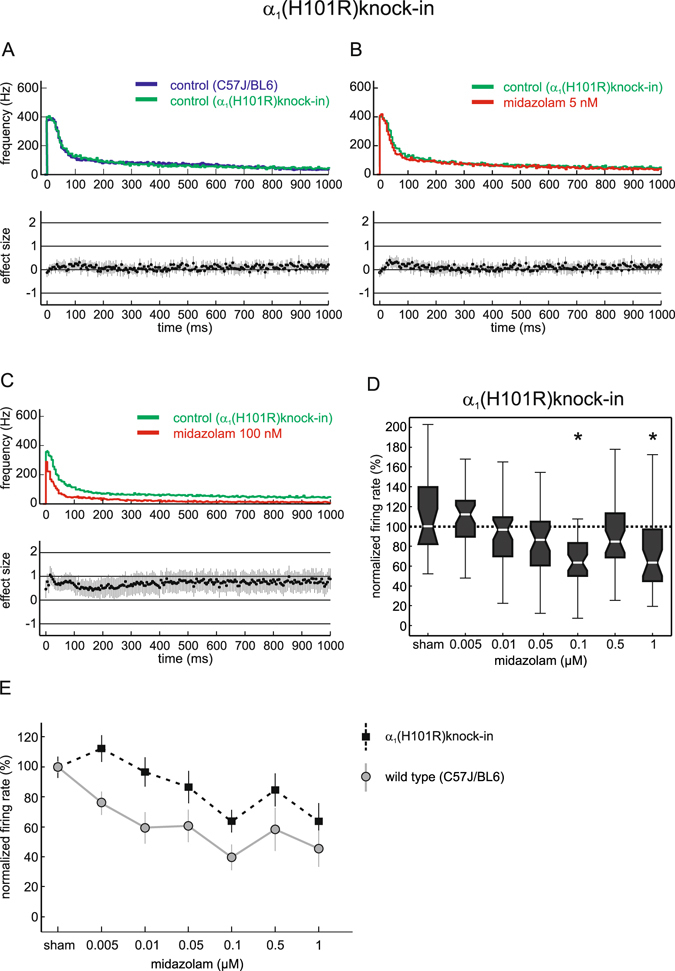
The actions of midazolam on global neuronal activity of organotypic slice cultures from GABA_A_ receptor α_1_(H101R)knock-in mice. (**A**) Comparison of neuronal activity of wild-type mice (dark blue) and α_1_(H101R)knock-in mice (green) under control conditions. No difference between the two genotypes could be detected. (**B**) Actions of 5 nM midazolam (red) versus control condition (green) in slices from α_1_(H101R)knock-in mice. Unlike in the wild-type mice, midazolam’s effect was absent in neocortical cultures from α_1_(H101R)knock-in mice. (**C**) Effect of 100 nM midazolam (red) versus control conditions (green). Only at this higher concentration midazolam depressed cortical activity also in cultures from α_1_(H101R)knock-in mice. (**D**) Depression of the overall action potential firing rate by midazolam in neocortical cultures from α_1_(H101R)knock-in mice. In contrast to wild-type slice cultures (see Fig. 1A) the spontaneous firing rate was significantly depressed only at 100 nM and 1 µM (*p < 0.001; Kruskal-Wallis test, post-hoc analysis (Bonferroni) followed by Mann-Whitney U test). (**E**) A comparison of the concentration-dependant actions of midazolam in neocortical cultures from wild-type mice (grey) and α_1_(H101R)knock-in mice (black) given as median and the 95% CI. Note that both curves run nearly parallel.

To test the impact of cortical α_1_-containing GABA_A_ receptors for midazolam we carried out experiments in cortical cultures derived from GABA_A_R α_1_(H101R) knock-in mice, in which α_1_-containing GABA_A_ receptors are insensitive to benzodiazepines, including midazolam^16^. The basal activity of cultures derived from α_1_(H101R)knock-in mice was 16.64 Hz; 14.87 (median, iqr, n = 136). This value was slightly higher than the value obtained with wild-type mice. However, when comparing PETH of the averaged up states, no differences could be detected between wild-type and α_1_(H101R) mice (Fig. 6A).

As can be seen in Fig. 6B,D, we were not able to determine any significant inhibitory effect in neocortical tissue cultures derived from α_1_(H101R)knock-in mice for concentrations of 5 nM and 10 nM midazolam, neither during up states nor for the overall action potential firing rate. Only at a concentration of 100 nM midazolam did induce significant depression during phases of high neuronal activity (Fig. 6C). The concentration response relationship (see Fig. 6D) illustrates that only 100 nM and 1 µM midazolam caused a significant reduction of the action potential firing rate (median 64%; iqr 33; p < 0.001; median 63%; iqr 52; p = 0.001; Mann-Whitney U test). When comparing the effects of midazolam in α_1_(H101R) mice to those seen in wild-type mice, we observed that the inhibitory action of midazolam once again did not increase steadily with increasing concentrations. The concentration response relationship curves nearly run parallel to each other (Fig. 6E).

## Discussion

Midazolam is metabolized into an active metabolite, 1-hydroxymidazolam. However, 1-hydroxymidazolam’s contribution to the neuronal depressant actions of midazolam is complex and still poorly understood^1–5^.

Within the central nervous system the neocortex seems to be the major target in mediating sedation by benzodiazepines^10, 17^. However, to date only little is known about the potentially differential effects of midazolam and 1-hydroxymidazolam on neuronal activity in this region of the brain. Therefore, in the present study we set out to compare the potency and characteristics of midazolam and 1-hydroxymidazolam to inhibit spontaneous cortical activity. We observed that midazolam, acting mainly via α_1_-containing GABA_A_ receptors, is by far more potent than 1-hydroxymidazolam and that midazolam and 1-hydroxymidazolam act quantitatively and qualitatively different.

Midazolam depresses cortical activity in a biphasic manner. This is in close agreement with results obtained from previous studies with diazepam^11, 14^. The most convincing hypothesis to explain this biphasic depression is the existence of at least two benzodiazepine binding sites, as proposed by Walters *et al*., leading to two separate mechanisms of benzodiazepine action^14^.

The first phase of midazolam’s action (steady state of depression) is in line with previous studies^18, 19^ and this “ceiling effect” could be one of the reasons for the clinical safety of benzodiazepines. There are, however, several possibilities to explain this finding: First, a saturation at the classical benzodiazepine site^11^ and second, a novel binding site for benzodiazepines at the GABA_A_ receptor, preventing further depression, as suggested in a previous study by Baur *et al*.^20^.

In accordance with previous clinical investigations and *in vivo* studies showing that sedation and changes of EEG frequencies could be induced by 1-hydroxymidazolam alone^1, 2, 21^ we found that 1-hydroxymidazolam inhibits neocortical network firing.

When comparing midazolam with 1-hydroxymidazolam it is evident that 1-hydroxymidazolam is far less potent. Inhibitory actions of 1-hydroxymidazolam could be observed starting at a concentration of 100 nM, corresponding to a plasma concentration of nearly 40 ng/ml. Considering the plasma concentration measured in a previous study^3^, 1-hydroxymidazolam may, therefore, contribute to the effects of midazolam, above all, in cases of deep sedation or in patients with severe renal dysfunction^22^. A summary of clinical and experimental findings concerning effects of midazolam and 1-hydroxymidazolam at different concentrations can be found in Table 1.

**Table 1:** Predicted concentrations of midazolam and 1-hydroxymidazolam in the brain, corresponding to the level of sedation.

Predicted concentrations in the brain	Light sedation	Profound sedation	Induction of general anesthesia
*midazolam* + *1-hydroxymidazolam*	*50* *nM* + 30 nM^1^	*670–2440* *nM* + 100–270 nM^3^	*2400* *nM* + 200 nM^42^
*1-hydroxymidazolam alone under experimental conditions*	115–230 nM^1^ 500 nM in rats^2^	no data	no data

The second row summarizes plasma concentrations taken from clinical studies, where 1-hydroxymidazolam is a product of metabolism of midazolam. The third row reports data where 1-hydroxmidazolam was given alone, i.e., in the absence of midazolam.

Meanwhile, a study by Bremer *et al*. demonstrated in critically ill patients plasma concentrations ranging from 60 to 136 ng/ml for midazolam and from 11 to 62.5 ng/ml for 1-hydroxymidazolam for lightly sedated patients as well as plasma concentrations ranging from 307 to 1106 ng/ml for midazolam and from 41 to 119 ng/ml for 1-hydroxymidazolam for deeply sedated patients^3^. Taking into account the molecular weights of the two compounds, midazolam’s free unbound fraction of approximately 3%^1^ and the brain-to-unbound-serum concentration ratio of almost 34^43^, this would correspond to a midazolam concentration of about 130 to 2,400 nM in the central nervous system. In the case of 1-hydroxymidazolam, the free unbound fraction is supposed to be 10%^1^ and the brain-to-unbound-serum concentration ratio around 8^43^. Thus, the concentration of 1-hydroxymidazolam in the central nervous system should range from 25 to 270 nM. These assumptions are in accordance with data from the literature, indicating that nanomolar concentrations of midazolam *in vitro* are of clinical relevance^18^.

In addition, we detected that midazolam administered at the low concentration of 5 nM caused a significant inhibition of the cortical network activity in cultures from wild-type, but not from α_1_(H101R)knock-in mice. Therefore it seems reasonable to assume that midazolam at low nanomolar concentrations predominantly acts via α_1_-containing GABA_A_ receptors. Thus, despite being a non-GABA_A_ receptor subtype-selective benzodiazepine^23^, midazolam’s sedative actions are largely mediated by α1-containing GABA_A_ receptors. In contrast to diazepam, at recombinant receptors midazaolam displays a preference for α1-containing GABA_A_ receptors over α2-containing GABA_A_ receptors: a recent study showed an approximately two-fold larger potentiation of GABA-induced currents by midazolam at GABA_A_ receptors containing α_1_-subunits compared with GABA_A_ receptors containing α_2_-subunits, whereas diazepam potentiated both receptor subtypes to a similar extent^16^.

To date, α_1_-containing GABA_A_ receptors have been shown to be of major importance for mediating sedation, amnesia and anticonvulsant activity of benzodiazepines^16, 24^. Due to midazolam’s high efficacy at this GABA_A_ receptor subtype, changes in quantity or distribution of this receptor subtype might have some clinical implications. For example, there is growing evidence that inflammation changes GABA_A_ receptor composition in the brain. Pribiag and Stellwagen demonstrated that TNFα decreases inhibitory synaptic strength by downregulation of cell-surface levels of GABA_A_ receptors^25^. In addition, interleukin-1β increases surface expression of α_5_-containing GABA_A_ receptors and a tonic current generated by these receptors, resulting in memory deficits^26^. Furthermore, it is known that assembling, trafficking, clustering and endocytosis of GABA_A_ receptors are highly dynamic and, thus, can be influenced by other factors like repeated seizures or alcohol abuse^27^. These changes in GABA_A_ receptor populations might well be contributing to the high variability of duration and the different qualities of sedation with midazolam. Considering that midazolam shows a preference especially for α_1_-subunit containing GABA_A_ receptors, small changes in the cell surface concentration of this GABA_A_ receptor subtype may have major implications for midazolam’s clinical actions.

Moreover, there is evidence indicating that the addictive properties of benzodiazepines are mediated via α_1_-containing GABA_A_ receptors^28, 29^. Finally, α_1_-subunits containing GABA_A_ receptors are involved in developmental plasticity. The present *ex vivo* results raise questions regarding the potential induction of undesired long-term changes by midazolam in immature neuronal networks^30^.

In summary, midazolam depresses cortical networks at the low concentration of 5 nM, predominantly mediated via α_1_-containing GABA_A_ receptors. This GABA_A_ receptor subtype is the major molecular target of benzodiazepines for sedation. Small changes of this receptor population, e.g., due to inflammatory diseases, may therefore serve as an explanation for the large variability of midazolam’s clinical properties.

Midazolam’s main metabolite, 1-hydroxymidazolam, is far less potent than midazolam itself. The qualitatively different actions of 1-hydroxymidazolam compared with those of midazolam might argue for differential affinities of these two drugs at the α1 GABA_A_ receptor subtype, possibly also at different GABA_A_ receptor subtypes, as has recently been described for clobazam and its metabolite N-desmethyl-clobazam^31^.

We conclude that when considering the clinically relevant concentrations of midazolam, used for sedation in intensive care medicine, midazolam is clearly more potent than its metabolite, i.e. the major clinical effects are caused by the parent drug midazolam itself, presumably acting on cortical neurons. The higher potency of 1-hydroxymidazolam seen in some *in vivo* studies could be due to other types of GABA_A_ receptors in lower brain structures, i.e. δ-subunit containing GABA_A_ receptors.

## Methods

### Organotypic slice cultures

All procedures were approved by the Animal Care Committee (Eberhard Karls University, Tübingen, Germany) and were in accordance with the institutional and federal guidelines of the German Animal Welfare Act (TierSchG). Wild-type C57BL/6J mice and GABA_A_ receptor α_1_(H101R)mutant mice on C57BL/6J background^15^ of both sexes were used. Neocortical organotypic slice cultures were prepared from three to five day old mice as described before^32^. In brief, animals were deeply anaesthetized with isoflurane and then decapitated. Cortical hemispheres were removed aseptically and 300 µm thick coronal slices were cut. Slices were fixed on glass coverslips by means of a plasma clot, transferred into plastic tubes containing each 750 µL of nutrition medium, to be then incubated in a roller drum at 37 °C. After 1 day in culture, antimitotics (10 µM 5-fluoro-2′-deoxyuridine, 10 μM ARA-c and 10 μM uridine) were added. The suspension was renewed twice a week.

### Electrophysiology

Extracellular multi-unit recordings were performed in a recording chamber mounted on an inverted microscope. Slices were perfused with artificial cerebrospinal fluid (ACSF) consisting of (in mM) NaCl 120, KCl 3.5, NaH_2_PO4 1.13, MgCl_2_ 1.0, NaHCO_3_ 26, CaCl_2_ 1.2, and glucose 11; then bubbled with 95% oxygen and 5% carbon dioxide. All experiments were conducted at 34 °C. For this purpose, a heating wire was glued onto a metal frame of the recording chamber and then heated using a direct current. ACSF-filled glass electrodes with a resistance of 3 to 5 MΩ were advanced into the tissue until extracellular multi-unit spike activity exceeding 100 µV in amplitude was visible. Data were acquired with the digidata 1200 AD/DA interface and AxoScope 9 software.

Intracellular voltage clamp recordings were performed on visually identified pyramidal neurons at room temperature. Pipettes were filled with a solution containing (in mM) 121 CsCL, 24 CsOH, 10 HEPES, 5 EGTA, 1 MgCl_2_ and 4 ATP, adjusted to pH 7.2 with 1 N HCl. Cells were voltage-clamped at −70 mV. To suppress glutamatergic synaptic transmission, we added 25 µM D-L-2-amino-5-phosphonopentanoic acid, 10 µM 6-cyano-7-nitroquinoxaline-2.3-dione and 1 µM tetrodotoxin. For the detection of tonic currents 20 µM bicuculline was added to the ACSF. Analysis of tonic currents followed the method proposed by Glykys and Mody^33^ and is described in detail in reference^34^.

### Preparation and Application of Test Solutions

Midazolam and 1-hydroxymidazolam were dissolved in dimethyl sulfoxide to a 1 mM stock solution. Flumazenil was dissolved in 99% ethanol to a 5 mM stock solution. Drugs were diluted in ACSF to the tested concentration and filled into glass syringes immediately before the experiment was conducted. The drug containing ACSF was applied via bath perfusion using syringe pumps, connected to the experimental chamber via Teflon tubing. When switching from ACSF to drug-containing solutions, the medium was replaced by at least 95% within two minutes. To ensure steady-state conditions, recordings were conducted 12 minutes after commencing the change of the perfusate. Previous studies have demonstrated that this time interval is sufficient for steady-state conditions^35, 36^ because diffusion times in slice cultures are considerably shorter compared to acute slice preparations^37, 38^.

### Data Analysis

Extracellular recorded signals were filtered and counted offline using self-written Matlab (R2008b) routines. The activity pattern of neocortical slice cultures was characterized by phases of spontaneous action potential firing (up states) separated by periods of neuronal silence. Action potentials were detected and the average firing rate was computed, using an automated event detection algorithm with a threshold set approximately 2 times higher than baseline noise. An up state was defined by an initial inter-event interval of 75 ms and a preceding silent period of at least 500 ms. All automated up state detection was checked by visual inspection based on field potentials. Parameters are shown as relative change compared to ACSF sham application. Each data set was checked for normal distribution by using qq plots. However, the hypothesis that data sets were normally distributed had to be rejected for the majority of these data sets. Therefore, grouped data are expressed as boxplots (line: median, box: lower quartile = 25^th^ percentile and upper quartile = 75^th^ percentile; whisker: 1.5*iqr (interquartile range); iqr: difference between the upper and the lower quartiles). For statistical analysis of extracellular detected neuronal spiking activity, we performed a combination of a non-parametric analysis of variance (Kruskal-Wallis test) and a multiple comparison post-hoc analysis (Bonferroni), followed by a Mann-Whitney U test to check for significant differences between the two groups.

To illustrate changes of the network firing, especially during phases of high neuronal activity (up states) peri-event time histograms (PETH) were calculated. For this purpose, the up states of every experimental condition were collected, divided into bins of 10 ms, and averaged. Because the length of up states was variable, the PETH were constrained to the first 1000 ms. To test for statistically significant effects, the effect size (Hedges’ g including the 95% confidence interval) was calculated. The 95% confidence interval excluding zero indicates significance at p < 0.05. A general rule of thumb says that absolute Hedges’ g values >0.8 indicate a strong effect and values >0.5 indicate a medium effect.

### Statistical comparison of the actions of midazolam and 1-hydroxymidazolam

An extra sums-of-squares F test^39^ was performed to statistically compare IC_50_ values of the concentration-response relations of normalized firing rates for midazolam and 1-hydroxymidazolam. The underlying null hypothesis was that both data sets (midazolam and 1-hydroxymidazolam) stemmed from concentration-response relations with the same IC_50_. Briefly, Hill functions with three and two free parameters (‘full’ and ‘constrained’ models, respectively) were each fitted to each data set. In the constrained model, the IC_50_ (Kd) value was set to a fixed value, determined from a full Hill fit to the union of both data sets. An F value was computed as F = (SS1-SS2)/(DF1-DF2)/(SS2/DF2) where SS1 is the sum-of-squares of the constrained model (summed for both data sets), DF1 the degrees of freedom of the constrained model (again summed for both data sets), and SS2 and DF2 the equivalent values of the full model. From this F value (with DF1-DF2 and DF2 degrees of freedom for the numerator and denominator, respectively) a p value was computed, which specified the probability of obtaining data sets at least as extreme as the measured samples, given the null hypothesis.

As midazolam and 1-hydroxymidazolam altered up state length in qualitatively different ways, a fitting approach for statistical comparison of both drugs’ effects was not deemed reasonable. Instead, we opted for a two-way analysis of variance (ANOVA) with drug type (midazolam and 1-hydroxymidazolam) and drug concentration as the independent factors, focusing on their interaction. We computed both the p-value and eta squared, a measure of effect size, for the interaction of drug type with drug concentration. 95% confidence intervals of eta squared were computed by bootstrapping. Prior to analysis via ANOVA, the data were Box-Cox-transformed^40^ as the distribution of normalized burst length often had long tails towards high values: for each concentration of both drugs, the transformation parameter lambda was estimated; from the resulting collection of lambda values an average weighted by the number of samples in each group was computed (lambda = 0.214) and all data transformed with this value.

All analyses were implemented in Matlab (R2016b) and partly made use of the measure of effect size toolbox^41^.
